# COL1A2 (p.Gly322Ser) Mutation Causes Late-Onset Osteogenesis Imperfecta: A Case Report

**DOI:** 10.7759/cureus.30172

**Published:** 2022-10-11

**Authors:** Hector Muñoz-Miro, Elyette Lugo, Simon Carlo, Norman Ramírez

**Affiliations:** 1 Surgery, Universidad Central del Caribe, Bayamon, USA; 2 Pediatrics, Mayaguez Medical Center, Mayaguez, USA; 3 Biochemistry, Ponce Health Science University, Ponce, USA; 4 Surgery, Ponce Health Science University, Ponce, USA; 5 Pediatric Orthopedics Surgery, Mayaguez Medical Center, Mayaguez, USA

**Keywords:** genetic mutation, spontaneous fractures, late onset, brittle bone disease, osteogenesis imperfecta

## Abstract

Osteogenesis imperfecta (OI) is a genetically inherited disorder that mainly affects the bones and causes a generalized decrease in bone mass. OI has a broad clinical spectrum ranging from the most severe form of OI which may cause in-utero death or stillbirth to the milder form. Clinical manifestations normally mitigate with an increase in age. We report a case of a healthy 12-year-old male who presented with a spontaneous fracture of the femur without trauma. The patient has no previous history of fractures, bone deformities or systemic conditions. The initial physical examination was unremarkable except for a bilateral subtle grayish sclera. Calcium, phosphorus, vitamin D, blood urea nitrogen (BUN), creatinine, and parathyroid hormone (PTH) values were within normal range. After genetic testing, the patient was diagnosed with OI due to a pathogenic COL1A2 (c.964G>A [p.Gly322Ser]) mutation. The first manifestation was at 12 years of age with a femur spontaneous fracture, which brings to the fact that the patient has a late onset of OI.

## Introduction

Osteogenesis imperfecta (OI), also called brittle bone disease, is a genetically inherited disorder that mainly affects the collagen type I and subsequently bones, cartilage and ligaments with a generalized decrease in bone mass [1]. The estimated prevalence of OI in the United States is approximately one in 15,000 to 20,000 births, categorizing it as an orphan disease [2]. Clinical manifestations include liability to fractures, skeletal deformities and extra-skeletal manifestations [3,4]. The hallmarks of skeletal deformities are short stature, vertebrae deformities, joint laxity, coxa vara, olecranon avulsion, and barrel-shaped chest [4]. These extra-skeletal manifestations include dental abnormalities (dentinogenesis imperfecta), progressive hearing loss, discoloration of the sclera, respiratory problems, cardiovascular diseases and neurological abnormalities [4,5].

OI results from mutations affecting COL1A1 or COL1A2, genes that encode the formation of pro-ɑ1(I), and ɑ2(I) chains in type 1 collagen, the main structural protein of pre-mineralized bone matrix [5,6]. Both chains contain a central triple helix, which is composed of uninterrupted repeats of Gly-X-Y tripeptide which give type 1 collagen stability [5-7]. Triple helix crosslinking proceeds normally if glycine is present in every third position [6,7]. The most common mutations of OI arise from the substitution of glycine by another amino acid in the crosslink, resulting in a defective type 1 collagen [6,7].

OI has a broad phenotypic spectrum ranging from the most severe form of OI which may cause in-utero death or stillbirth to the mild forms [3,5,6]. The diagnosis of OI is based on clinical findings, radiological findings or biochemical analysis via DNA-based sequencing of COL1A1 and COL1A2 [8]. In a primary care facility, the clinician should explore family history, medical history and physical examination [5]. The confirmatory resource is a genetic evaluation when the history is not determinant in the diagnosis. Commonly, manifestations are presented during infancy with a decrease or mitigation of symptoms as age increases [9]. We report the first case of late onset of OI, in which the first manifestations appear at 12 years of age.

## Case presentation

**Figure 1:**
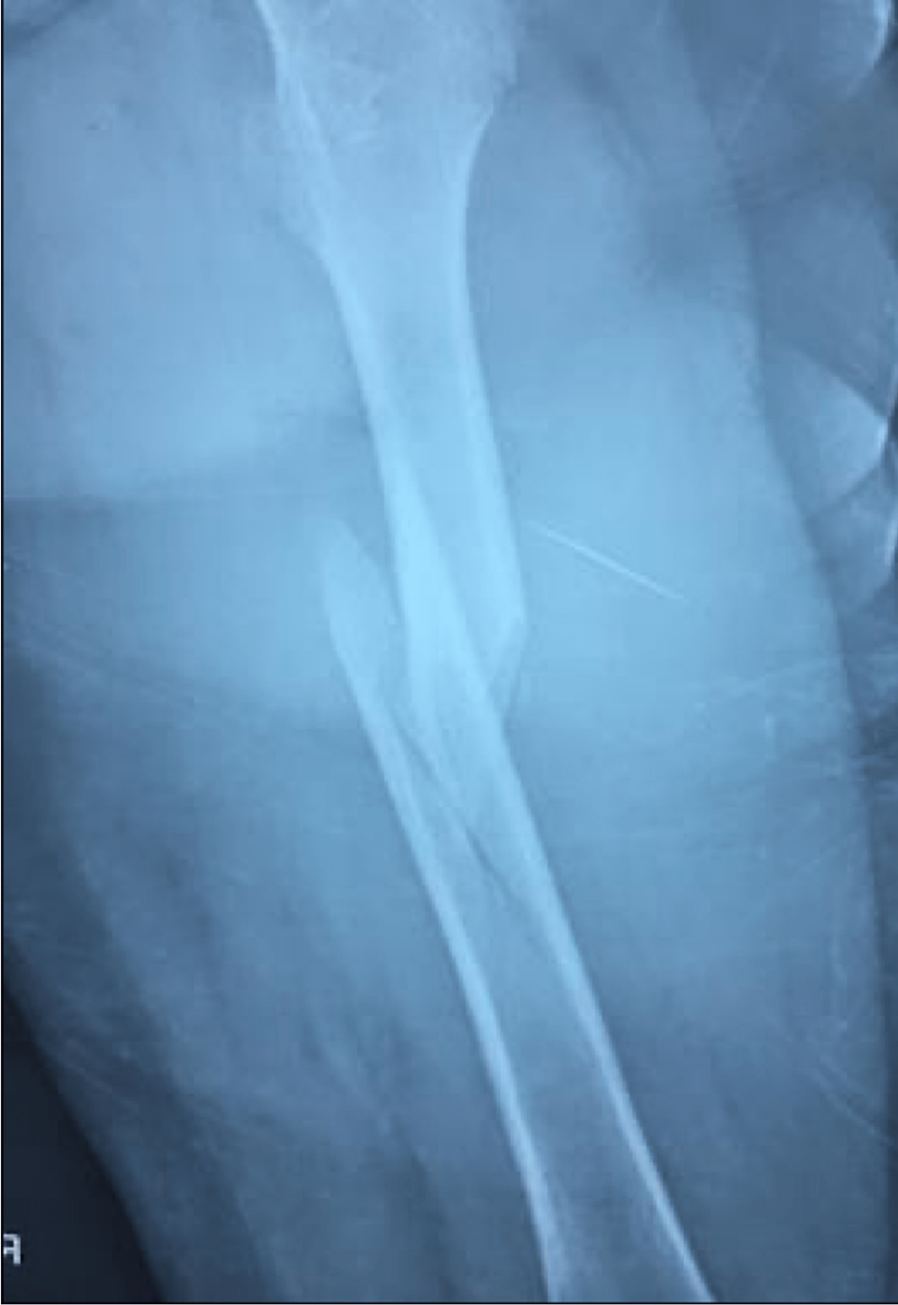
X-ray showing right femur fracture

**Figure 2:**
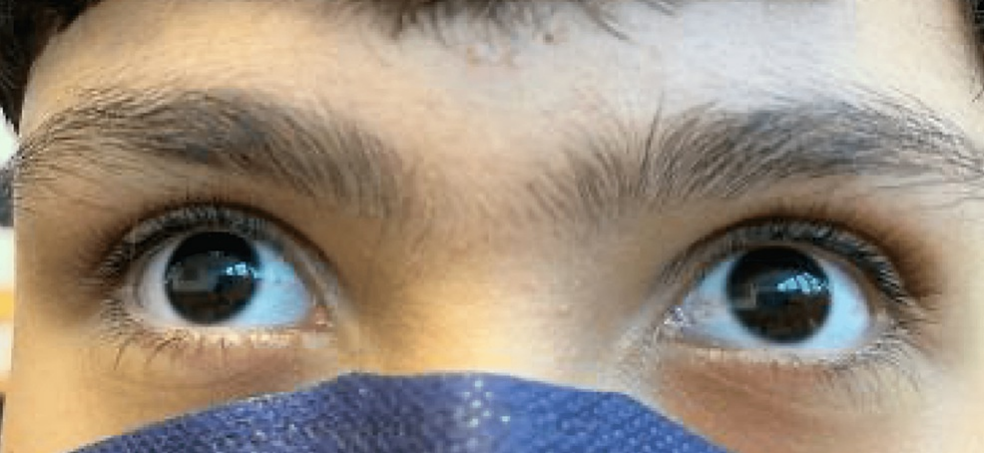
Bilateral subtle grayish sclera presentation on a 12-year-old male

**Figure 3:**
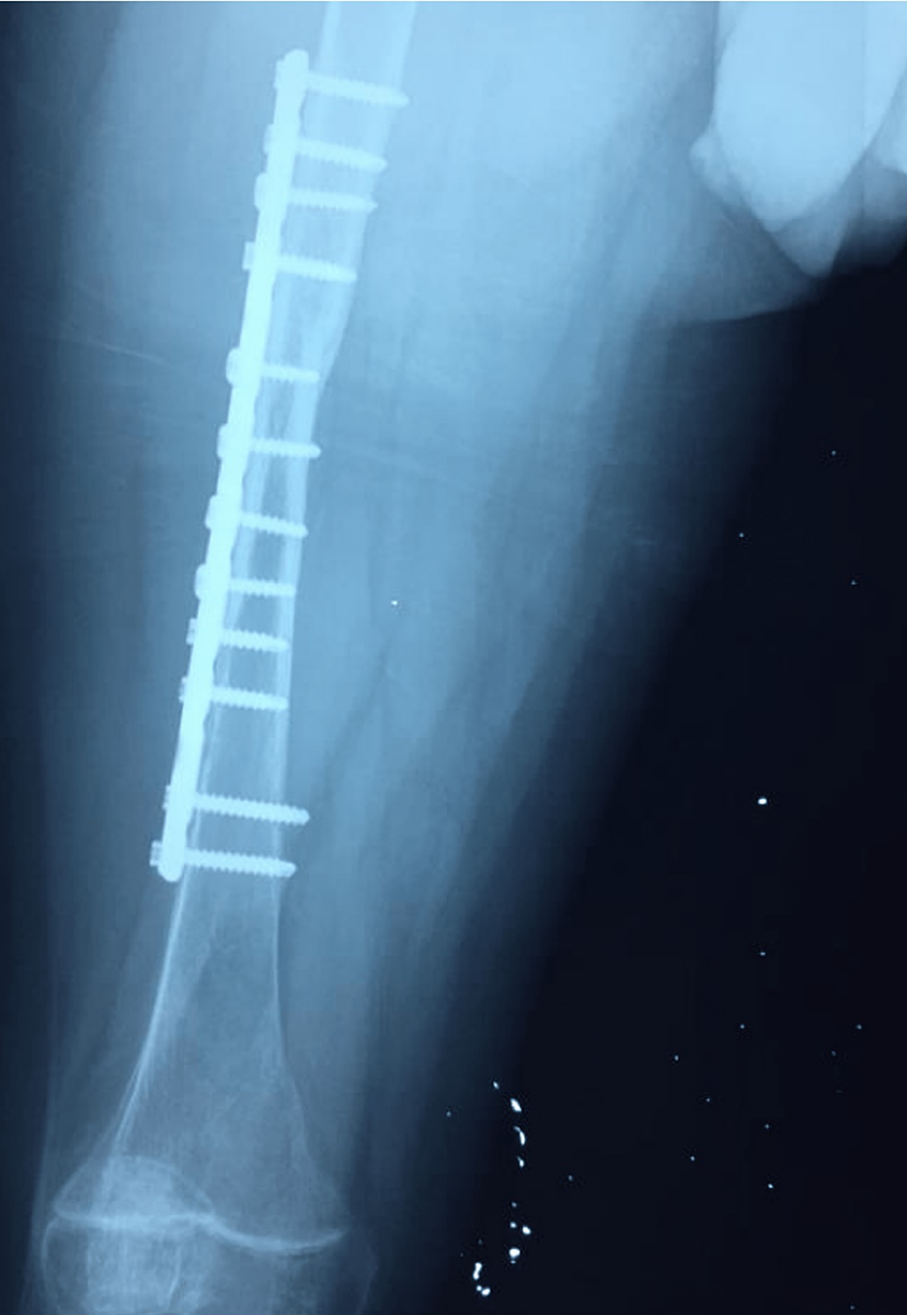
Post-operative x-ray of right femur open reduction internal fixation

**Figure 4:**
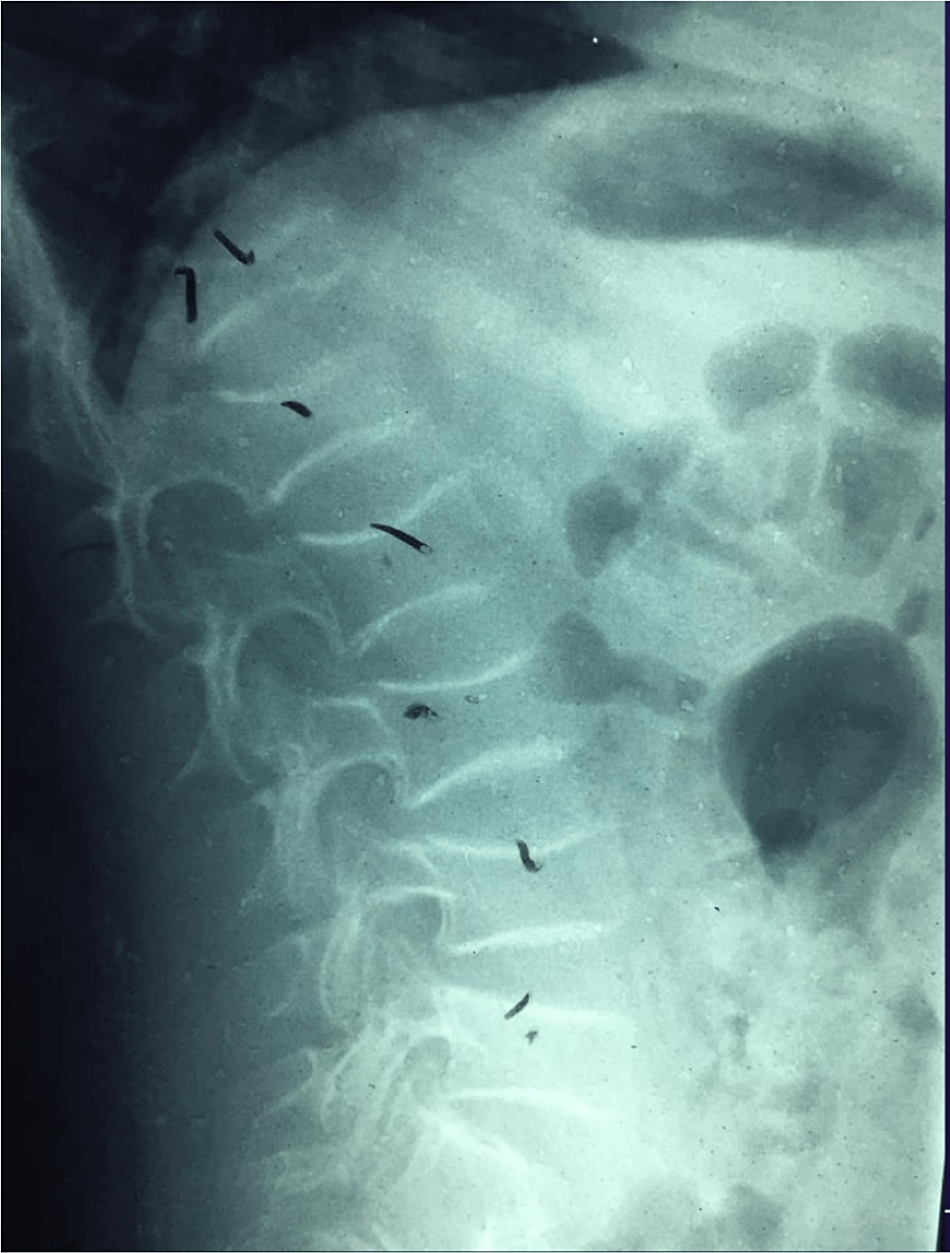
X-ray showing adjacent intervertebral discs compressions

A 12-year-old healthy male born to non-consanguineous parents of Puerto Rican descent was admitted to the emergency room due to a spontaneous right femur spiral fracture without trauma (Figure 1) and a left third distal radius fracture. The patient stated that he was walking and suddenly felt a sharp pain in the right femur that provoked a fall, from his body height, and fractured the third distal radius secondary to this fall. The patient specified that the right femur pain was before the fall. In his medical history, the patient had no previous history of fractures. Family history was significant for hypertension and diabetes mellitus. On physical examination (PE), his height was 150 cm, body weight: 59 kg, body mass index (BMI): 26.3 kg/m^2^, and blood pressure: 130/80 mmHg. PE showed an alert and oriented healthy male with swollen and tender left distal wrist and right thigh with adequate distal neurovascular status. Also, PE demonstrated bilateral subtle grayish sclera (Figure 2) without dental and audiology pathologies. Routine investigations revealed a completely normal hemogram and urinalysis. Vitamin D, creatinine, blood urea nitrogen (BUN), phosphate, alkaline phosphatase and parathyroid hormone (PTH) levels were all under the normal range. He underwent open reduction internal fixation with a bone plate and screws for the right femur fracture (Figure 3). The left distal third radius fracture was treated with close reduction and casting. This unaccountable femur fracture captured the attention of the orthopedic surgeon because of its puzzling origin. A bone survey was performed and revealed biconcave deformity of the vertebral body (Figure 4). An underlying pathology was suspected due to the clinic scenario and imaging findings, and genetic tests were ordered postoperatively to the proband, both parents and older sister. The genetic study revealed that the proband, his father and his older sibling were all positive for COL1A2 (c.964G>A [p.Gly322Ser]) gene and negative for COL1A1. Father and older sibling had never experienced OI symptoms. Mother was negative for both genetic markers. Based on the COL1A2 positive finding, an audiogram was ordered with negative results. Also, dual-energy x-ray absorptiometry (DEXA) was performed with a Z-score of -3.0, compatible with decreased bone mass. The cardiovascular evaluation was performed with no further clinical findings. OI was diagnosed based on the clinical radiologic and biochemical analysis findings; therefore, bisphosphonate treatment was started.

## Discussion

Mutations in COL1A2 are associated with collagen-related diseases found in most connective tissues and are abundant in bone, cornea, dermis and tendon. The proband presents with a pathogenic heterozygous variant of the COL1A2 gene (c.964G>A; p.Gly322Ser) on chromosome 7q21 who showed the first manifestations of OI at 12 years of age. The presented case has no previous events of fracture nor the expected manifestation of OI. However, the emergency room showed subtle grayish sclera and vertebral deformities.

Several cases have been reported as having late manifestations of OI. Still, an in-depth analysis of these cases reveals recurrent fractures during infancy, skeletal deformities, positive family history of OI, all of which would disqualify these as late-onset cases, instead being late diagnosed [3,5,10]. In 1992, Nicholls et al. reported a family with affected members presenting with late-onset fractures, compatible with a different type of OI Sillence classification [10]. However, all of them had blue sclera, a feature of OI, although the significant signs of short stature, joint hypermobility, skin laxity, and easy bruising suggested an initial diagnosis of Ehlers-Danlos syndrome [10]. In 2005, Strevel et al. reported a 24-year-old woman who presented with lower back pain after water tubing [3]. Type 1A OI was established after the analysis revealed a Colles fracture at 9 and several digital fractures in infancy [3]. Her father and sister were diagnosed with osteoporosis and multiple fractures, including a hip fracture [3]. Her PE revealed blue-gray sclera, scoliosis, and a murmur [3]. Delineating yet another case toward late diagnosis rather than late onset. Lastly, in 2008, Sezer et al. reported a 38-year-old female admitted to the clinic for lower back pain [5]. She was diagnosed with OI after her medical and family history revealed several visits to specialists for lower back pain, dental problems, headache, bilateral hearing loss since infancy, scoliosis, and temporomandibular joint subluxation [5]. Three of five of her children, her sister, brother, mother, and grandmother, had blue sclera, and her children had spontaneous fracture history [5]. Thus, emphasizing a late diagnosis of OI because of her early manifestations.

OI type I-IV is commonly associated with mutations in COL1A1 and COL1A2 with a lifespan depending on the type [2,4,6,7]. COL1A1 and COL1A2 are genes that encode the formation of pro-ɑ1(I), ɑ2(I) chains in type 1 collagen [4,6,7]. A defect in any of these genes could affect the quantity or structure of type I collagen [4,6,7]. Diseases related to mutations only in COL1A1 may include Caffey diseases, arthrochalasia type of Ehlers-Danlos syndrome (aEDS) type I, and osteoporosis in adulthood [11]. Mutations in COL1A1 are more susceptible and commonly express a disease because it codes for two of the three alpha chains that form collagen type I, and this explains why patients with a mutation in COL1A1 have severe symptoms [12]. When conferring only a mutation in COL1A2, aEDS type II, a cardiovascular form of Ehlers-Danlos syndrome and OI are among the possible disorders related to this mutation [11]. Patients with COL1A2 mutation should be evaluated for possible defects in cardiac valves in the long term since studies have suggested that it could take time to develop it [3]. In this case, the presented scenario showed an autosomal dominant inheritance with variable expression and incomplete penetrance of OI, which explained why the father and older sibling have not shown any manifestations [5].

## Conclusions

There are instances where the diagnosis of OI is not evident, and the only clinical feature is unexplained fractures. In these cases, molecular testing as DNA base sequencing of COL1A1 and COL1A2 is the best way to diagnose a possible underlying pathology. Collagen analysis should be the first line of genetic investigations in any individual presenting with spontaneous fractures and colored sclera as a late manifestation. Usually, the patient with OI is diagnosed at an early age and clinical manifestations normally mitigate with an increase in age. This case report brings to the medical community the possibility of OI patients debuting their pathological symptoms at adolescence, instead of an early age.
